# Serotonergic Modulation of Conditioned Fear

**DOI:** 10.6064/2012/821549

**Published:** 2012-10-09

**Authors:** Judith R. Homberg

**Affiliations:** Donders Institute for Brain, Cognition and Behaviour, Department of Cognitive Neuroscience, Radboud University Medical Centre, Geert Grooteplein 21, Route 126, 6525 EZ Nijmegen, The Netherlands

## Abstract

Conditioned fear plays a key role in anxiety disorders as well as depression and other neuropsychiatric conditions. Understanding how neuromodulators drive the associated learning and memory processes, including memory consolidation, retrieval/expression, and extinction (recall), is essential in the understanding of (individual differences in vulnerability to) these disorders and their treatment. The human and rodent studies I review here together reveal, amongst others, that acute selective serotonin reuptake inhibitor (SSRI) treatment facilitates fear conditioning, reduces contextual fear, and increases cued fear, chronic SSRI treatment reduces both contextual and cued fear, 5-HT_1A_ receptors inhibit the acquisition and expression of contextual fear, 5-HT_2A_ receptors facilitates the consolidation of cued and contextual fear, inactivation of 5-HT_2C_ receptors facilitate the retrieval of cued fear memory, the 5-HT_3_ receptor mediates contextual fear, genetically induced increases in serotonin levels are associated with increased fear conditioning, impaired cued fear extinction, or impaired extinction recall, and that genetically induced 5-HT depletion increases fear conditioning and contextual fear. Several explanations are presented to reconcile seemingly paradoxical relationships between serotonin levels and conditioned fear.

## 1. Introduction

Persistence of conditioned fear is a core process in the development of posttraumatic stress disorder (PTSD) as well as other anxiety-related disorders and depression [1]. An issue that is occupying scientists for decades is the phenomenon that some individuals more than others are vulnerable to engage into increased conditioned fear responses. Although lots of progress has been made in the understanding of the brain circuit implicated in conditioned fear, the factors that trigger differential function of this brain circuit remain rather elusive. Here it is my aim to highlight the role of serotonin in conditioned fear. Serotonin (5-HT) is an ancient molecule that may help the individual to attune behaviour towards specific elements of the environment, particularly conditioned stimuli. Indeed, it has been shown that 5-HT levels in the amygdala are increased in response to cued conditioned fear, but not unconditioned fear [2, 3], and that fear conditioning and fear-potentiated startle increase c-Fos expression in raphe nuclei [4]. Yet, there are several conditioning processes involved in the expression of conditioned fear, like fear conditioning, (re)consolidation, expression, extinction, and the recall of fear extinction. As understanding of the conditioning processes affected by 5-HT is extremely useful in, for example, the timing of antidepressant or anxiolytic treatment and the development of individualized behavioural cognitive therapies, it is my aim to provide an overview of human and particularly rodent fear conditioning studies manipulating the serotonergic system in various ways and to propose new views on the specific function of 5-HT. I used PubMed as tool to search literature and employed the key words “serotonin” and “fear conditioning.”

## 2. Behavioural Measures of Conditioned Fear

### 2.1. Fear Conditioning

**Figure 1:**
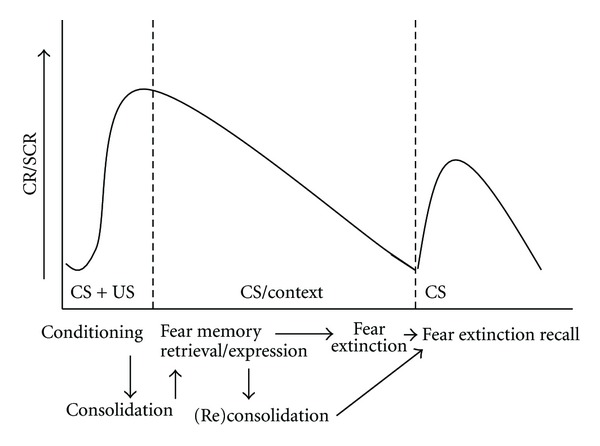
Sequential fear conditioning and extinction (recall) processes. CR: conditioned response; SCR: skin conductance response; US: unconditioned stimulus; CS: conditioned stimulus.

The paradigm that is typically used in the laboratory to measure fear *conditioning* involves the pairing of an initial neutral conditioned stimulus (CS) (such as a tone, light, and/or context) with a salient aversive unconditioned stimulus (US) (such as an electric footshock or loud noise) that reflexively evoke unconditioned fear responses (URs) (Figure 1). Through CS-US association formation, the CS acquires a predictive value and elicits conditioned fear responses (CRs), which are expressed as increased skin conductance responses (SCRs) in humans and conditioned freezing in rodents in case of shocks [5]. Freezing is characterized by immobility, the absence of any movement except for breathing, which is actually opposite to the acute behaviour response to footshock, which is jumping. Yet, given that there are no escape possibilities, immobility is an adaptive response [6]. After acquisition of the CS-US association, its consolidation takes places during the first 3 hours after fear conditioning, allowing strengthening of the memory trace through a cascade of intracellular signaling pathways [7]. Reexposure to the CS in the absence of the US, which is typically measured 24 hr after conditioning, triggers memory retrieval and allows the measurement of the *expression* of conditioned fear. This memory retrieval initiates two potentially dissociable but opposite processes: reconsolidation and extinction. *Reconsolidation* acts to stabilize, update, or integrate new information into long-term memories, whereas *extinction* (which increases with repeated unreinforced CS presentation) tends to weaken the expression of the original memory. The more conditioned freezing the animals show, the higher the fear memory is, albeit it is behaviourally indistinguishable whether this reflects increased reconsolidation or weak extinction. Importantly, extinction does not erase the CR memory but reflects new learning, an association between the CS and safety. Thus, the conditioned memory trace and the extinction memory trace coexist and compete, such that the extinction memory inhibits expression of the CR [8]. Besides the CS, also reexposure to the context in which fear conditioning took place can trigger the expression of conditioned fear. To dissociate the two, from this point and further on, CS-evoked fear is termed *cued fear*, and context-evoked fear is designated as *contextual fear*. Finally, unreinforced CS presentation after a delay triggers the *recall of the extinction memory*. Although conditioned fear-evoked behavioural and autonomic responses are adaptive (i.e., normal) components of the animal's defense mechanisms, they can become maladaptive when they are displayed out of context. For instance, when extinction is delayed upon repeated unreinforced CS presentation or when the recall of the extinction memory is impaired, the individual is at risk for anxiety-related disorders. 

### 2.2. Fear-Potentiated Startle

The fear-potentiated startle paradigm measures conditioned fear by an increase in the amplitude of a simple reflex (the startle reflex in response to sudden high-intensity noise bursts) in the presence of a cue previously paired with shock. In humans the fear-potentiated startle response is measured by electromyographic (EMG) recordings from the orbicularis oculi muscle, and in rodents by vertical movement. This paradigm offers the advantage that it is reflected by an enhancement rather than a suppression of ongoing behaviour [9]. Yet, in this paradigm the processes of fear (re)consolidation, extinction, and extinction recall are less dissociable compared to the fear conditioning paradigm. Using this paradigm two types of threat responses are dissociated, namely, phasic short-duration fear in response to fear cues and sustained long-duration anxiety induced by the context in which shocks have been given. The latter may model anticipatory anxiety, which is impaired in panic disorder [10].

## 3. The Neural Circuits Underlying Conditioned Fear

**Figure 2:**
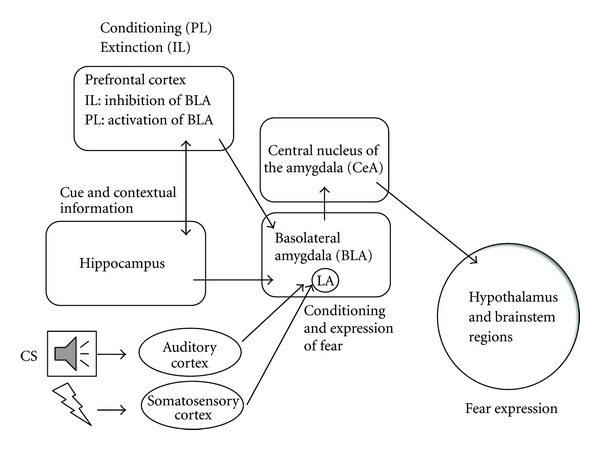
Simplified scheme of the neurocircuit mediating fear conditioning and extinction. PL: prelimbic cortex; IL: infralimbic cortex; LA: lateral amygdala.

Both human noninvasive neuroimaging studies and rodent invasive studies have revealed that the prefrontal cortex (PFC), hippocampus, and amygdala play a central role in fear conditioning and extinction (recall) (Figure 2). In general, the amygdala is thought to be involved in evoking fear and stress responses, the PFC exerts (inhibitory) control over the amygdala-mediated defensive behaviours, and the hippocampus signals the contextual associations of fear. During conditioning the CS is processed in the auditory cortex (if it is an auditory CS) and the US in the somatosensory cortex. These sensory cortices project to the lateral amygdala (LA), which is a critical site of CS-US convergence, associative plasticity, and memory storage. Following fear conditioning, the LA projects to the central nucleus of the amygdala (CeA), which elicits the fear response through the periaqueductal grey (PAG), hypothalamus, and brain stem. During fear extinction the ventromedial prefrontal cortex (vmPFC) is recruited. In rodents, the vmPFC can be subdivided into the infralimbic (IL) and prelimbic (PrL) regions which exert opposite roles; whereas the IL cortex inhibits the expression of fear, the PrL cortex facilitates the expression of fear. The IL cortex exerts its inhibitory role through inhibition of the basolateral amygdala (BLA) and the intercalated cell mass that project on their turn to the CeA. The PrL cortex, on its turn, stimulates the BLA and CeA and thereby enhances fear (e.g., [11]). The hippocampus comes into play when contextual fear is triggered and helps the PFC in the formation of the CS-US memory [12, 13]. Taken together, the idea is that during acquisition of extinction a novel memory is formed through hippocampus—vmPFC—BLA interactions, which, following consolidation, leads to a hippocampus-dependent vmPFC inhibition of the CeA, preventing the CR. Exactly this mechanism is impaired in PTSD [14, 15]. 

The neural mechanisms underlying fear potentiated startle overlap but are somewhat different. The sensory information is derived from the auditory nerve, cochlear nucleus, and the ventrolateral lemniscus, which provide input to the CeA. The CeA, on its turn, projects to the pontine reticular formation and excitates spinal motoneurons that give rise to the startle response [16]. However, this pathway may contribute to short-duration cue-induced fear. Long-duration context-induced anxiety, on the other hand, may be more diffuse and mediated by projections from the BLA and lateral CeA to the bed nucleus of the stria terminalis (BNST) [17–20]. The hypothesis that the BNST is implicated in long-duration sustained context-dependent anxiety corresponds to results showing a role of the BNST in context conditioning [19]. 

Because the amygdala, the BNST, and other forebrain structures are innervated by the raphe nuclei (where the serotonergic cell bodies are located) [21] and because 5-HT is implicated in affect and mood [22], serotonergic drugs have gained substantial interest in stress, fear conditioning, and clinical studies.

## 4. The Serotonergic System

5-HT in animals is derived from the essential amino acid L-tryptophan and transformed by the rate-limiting enzyme L-tryptophan-hydroxylase (Tph) into 5-hydroxytryptophan (5-HTP) [23]. The enzyme 5-hydroxy-tryptophan-decarboxylase removes a carboxyl group to form the final product to generate 5-HT. Like other neurotransmitters, 5-HT is transported into the vesicles near the presynaptic membrane of neurons by the vesicular monoamine transporter (Vmat). Upon fusion of the vesicle with the cell membrane, 5-HT is released into the synaptic cleft, where it can diffuse and bind to receptors to one or more of the 16 5-HT receptors that have been identified in the mammalian brain: 5-HT_1A,1B,1C,1D,1E,1F_, 5-HT_2A,2B,2C_, 5-HT_3A,3B_, 5-HT_4_, 5-HT_5A,5B_, 5-HT_6_, and 5-HT_7_. All of them are G-protein coupled receptors, except for the 5-HT_3A,3B_ receptors, which are ligand-gated ion channel receptors [24, 25]. In rodents, the presynaptic 5-HT_1B_ receptor autoregulates 5-HT release, while in humans it is the 5-HT_1D_ receptor that fulfils this purpose [25]. The presynaptic 5-HT_1A_ receptor is located in the raphe nuclei and regulates the firing of serotonergic raphe neurons that project to widespread regions in the brain as well as to the spinal cord. The 5-HT_1A_ and 5-HT_1B/D_ receptors, as all other 5-HT receptors, are also found postsynaptically and mediate a myriad of signalling pathways. Whereas there are 17 5-HT receptors, there is a single transporter responsible for the reuptake of 5-HT, which is termed the 5-HT transporter (5-HTT) [26]. Degradation of 5-HT in the brain is mediated by monoamine oxidase A (MAOA), with the resulting product being 5-hydroxyindoleactic acid (5-HIAA). In sum, the serotonergic system is composed of many different components and can be influenced by the amount of tryptophan in food, variation in serotonergic genes, or pharmacological agents that affect synthesis/degradation enzymes, 5-HT receptors, or 5-HTT. 

5-HT is a phylogenetically ancient neurotransmitter and is implicated in many central functions. These include control of mood, sleep, cognitive functions, learning and memory, and ingestive and reward-related behaviour. It is therefore not surprising that 5-HT is implicated in virtually all psychiatric and neurological disorders. Despite decades of research the precise function of serotonin is still unclear. 5-HT is well known for regulating sensory information processing and motor responses, but perhaps most challenging (and also most relevant here) is the understanding of its role in the regulation of emotion and cognitive processes. Classical ideas about the function of 5-HT involve those of aversive processing [27] and behavioural inhibition [28]. Whereas these functions may appear unrelated, they can be reconciled by considering motivation and activation as distinct, but connected, behavioural domains. That is, increased aversive processing (motivational domain) is likely to lead to behavioural inhibition (activational domain) [29]. As an example, acute tryptophan depletion (ATD), lowering central 5-HT levels, increases punishment prediction, suggesting that 5-HT promotes the inhibition of responses to punishing outcomes. 5-HT-mediated increased inhibition of aversive thoughts may allow individuals to reduce the impact of aversive events [30]. An alternative, evolutionary, view is that 5-HT increases the sensitivity to environmental stimuli. A classical finding is that the short (s)-allelic variant of the common serotonin transporter polymorphism (5-HTTLPR) is associated with anxiety-related traits [31]. Since the 5-HTT is responsible for the reuptake of 5-HT, individuals carrying the s-allele (associated with decreased transcription of the 5-HTT gene) may exhibit increased extracellular 5-HT levels. Evidence, however, is lacking. Positron emission tomography (PET) studies could not reveal changes in 5-HTT binding in 5-HTTLPR s- versus l-allele carriers [32, 33]; but see [34], which has raised the idea that the 5-HTTLPR affects neurodevelopmental processes. This idea is supported by evidence that 5-HT has a critical role in cell division, neuronal migration, differentiation, and synaptogenesis [24, 35]. Functional brain imaging studies have indeed shown structural and functional changes in, amongst others, the amygdala and PFC, which resemble brain phenotypes found in depressed patients [36–38]. Yet, also evidence for this neurodevelopmental view is lacking, which complicates the interpretation of 5-HTTLPR effects in terms of 5-HT levels. Despite that the precise function of 5-HT is not fully clear, it is clear that it modulates the effects of valenced stimuli.

## 5. Selective Serotonin Reuptake Inhibitors

Selective serotonin reuptake inhibitors (SSRIs) are well known to increase 5-HT levels through inhibition of the 5-HTT. They are used in the treatment of depression but also have known anxiolytic effects [39, 40]. Their broad use is attributed, in part, to the absence of side effects [41]. Several types of SSRIs have been synthesized, which differ in their 5-HTT selectivity. The most widely used (and known) ones are citalopram, escitalopram, fluoxetine, fluvoxamine, paroxetine, and sertraline. Clinically (depressive) symptoms initially worsen, and alleviation of depressive symptoms is only seen after chronic SSRI treatment for about 3–6 weeks. The explanation for the initial worsening of symptoms has been related to the activation of inhibitory 5-HT_1A_ autoreceptors in the raphe nuclei, leading to a reduction in the firing rate of the serotonergic raphe neurons. Yet, in terminal regions, this would be compensated by increases in 5-HT levels through 5-HTT inhibition. Upon repeated exposure to SSRIs, these receptors desensitize, which leads to disinhibition of the firing of raphe neurons [42]. Although this hypothesis is supported by several lines of evidence, there are also ideas that the delayed therapeutic effects of SSRIs are due to neuroplastic changes, which need time to develop [43]. 

### 5.1. Effects of SSRIs on Fear Conditioning

Evidence for differential effects of acute and chronic SSRI treatment on fear conditioning was provided by Burghardt et al. [44] who found that acute citalopram administration enhanced fear conditioning, whereas chronic treatment (21 days) reduced the acquisition of auditory fear conditioning. In comparison, treatment with tianeptine (serotonin reuptake enhancer) had no effect acutely but also reduced the acquisition of tone conditioning when administered chronically. Perhaps there is an inverted U-shaped relationship between 5-HT levels and fear conditioning, such that low and high 5-HT levels both decrease fear conditioning. 

Montezinho and coworkers [45] tested the acute effects of the most selective SSRI, escitalopram, and found that the acquisition of contextual fear conditioning was significantly increased when the drug was applied before the acquisition. However, when escitalopram was applied after CS-US conditioning just prior to the conditioning test (i.e., the fear memory retrieval test), the expression of contextual fear was decreased. And when administered during memory consolidation, that is, immediately after CS-US acquisition, escitalopram dose-dependently enhanced conditioned contextual freezing. Given that escitalopram (at a dose that affects memory consolidation) increased hippocampal 5-HT levels fourfold without changing dopamine or noradrenaline levels [45], it is possible that increased 5-HT levels in the hippocampus facilitated conditioning and its consolidation, but impaired its retrieval. Another possibility is related to the fact that the acute response to footshock is expressed as jumping, which is opposite to the freezing CR the animals show when presented to the context without footshock. It may be that the CR that was acquired under influence of 5-HT involved jumping, which interfered with the conditioned freezing response. 

### 5.2. Effects of SSRIs on Conditioned Fear Expression

Regarding fear expression, as measured by CS or context exposure during the memory retrieval test, it was found that acute citalopram treatment did not alter baseline responding but significantly increased cued fear-potentiated startle, whereas the effect of acute citalopram on sustained (context-induced) fear was milder [46]. A different pattern was found when citalopram was tested chronically. Chronic citalopram-treated human subjects did not differ from placebo-controlled individuals for baseline startle and short-duration fear-potentiated startle measures but displayed reduced long-duration context-dependent startle potentiation under predictable (i.e., cued) conditions [46, 47]. These results show that whereas acute citalopram can exacerbate the expression of fear, chronic citalopram has anxiolytic effects. Furthermore, the data show that cued fear and contextual fear are mediated by distinct neural systems. 

In rodents, Muraki and colleagues [48] reported that acute citalopram (applied 24 hours after conditioning and 4 hrs before the test) significantly reduced contextual fear. Burghardt et al. [49] showed that, after drug-free fear conditioning, a single pretesting injection of the SSRI citalopram or fluoxetine significantly increased cued fear. There was no effect of the antidepressant tianeptine (a serotonin reuptake enhancer) [50] or the norepinephrine reuptake inhibitor tomoxetine, suggesting that this effect is specific to SSRIs. Interestingly, as was reported in humans, these two studies suggest that acute SSRI treatment has opposite effects on cued and contextual fear. Also Hashimoto et al. [51], Nishikawa et al. [52], and Santos et al. [53] showed that pretesting SSRI treatment decreased freezing to a fear-conditioned context. Furthermore, Hashimoto and coworkers [54] showed that an acute citalopram challenge reduced contextual freezing and that repeated citalopram injection twice daily for 7 days diminished this effect. However, no changes in contextual freezing were observed in response to chronic fluoxetine treatment (3 weeks) [55], which may be explained by the type of SSRI. Nonetheless, there seems to be rather high consensus that acute SSRI treatment exerts opposite effects on cued and contextual fear. This may be attributable to differences in the neural switches that are recruited by these tasks and their respective serotonergic inputs. It may be that serotonergic effects on the CeA (involved in cued fear) and BNST (involved in contextual fear) differ, although no studies are available to support this option. It has also been reported that animals bearing median raphe electrolytic/NMDA lesions or microinjections of the 5-HT_1A_ receptor agonist 8-OH-DPAT into the median raphe showed a significant decrease in time spent in contextual conditioned freezing, but an increase in cued fear-potentiated startle [56]. Because the hippocampus is innervated by the median raphe nucleus and the amygdala by the dorsal raphe nucleus [57, 58], it is possible that median raphe inactivation reduces the retrieval of hippocampus-dependent contextual information, whereas the same hippocampal inhibition may disinhibit the amygdala and thereby increase the reconsolidation of the amygdala-dependent CS memory [59].

### 5.3. Effects of SSRIs on Conditioned Fear Extinction

Finally, it has been demonstrated that chronic fluoxetine treatment prevented the reemergence of cued and contextual freezing when reexposed to three tone-shock pairings after fear extinction. This suggests that chronic fluoxetine treatment favors extinction memory by dampening the reactivation of the original tone-shock association [60]. Favoring extinction memory was also demonstrated by Karpova et al. (2011) [61] who showed that chronic SSRI treatment erased fear, but only when combined with extinction training. These data show that the synergy between pharmacotherapy and behavioural cognitive therapy is needed to erase fear memories, which do not reemerge during renewal or spontaneous recovery [61]. The rationale behind this might be that SSRIs are only effective when neurons are activated. That is, SSRIs increase extracellular 5-HT levels by inhibiting the 5-HTT, but if the neuron is not releasing 5-HT, this inhibition has little effects. Increases in extracellular 5-HT levels in the brain areas that are stimulated by extinction training, such as the BLA, hippocampus, and IL cortex, leads to neuroplastic changes, which in this study were reflected by a more juvenile state of perineuronal nets. This may have contributed to the erase of the fear memory. Furthermore, also changes in synaptic plasticity were found in the external capsula [61]. As the authors concluded, increased cortical input and synaptic plasticity in LA may facilitate associational learning in specific extinction circuits or suppress fear expression by recruiting inhibitory interneurons that project to the CeA. Finally, the 129S1/SvImJ (S1) inbred mouse strain exhibited fear overgeneralization to ambiguous contexts and cues, impaired context extinction, and impaired safety learning, relative to the (good-extinguishing) C57BL/6J (B6) strain. Fear overgeneralization and impaired extinction, which were related to downregulation of negative feedback control of the hypothalamic-pituitary-adrenal axis, were rescued by chronic fluoxetine treatment [62]. Because fluoxetine normalized S1 fear to B6 levels on the postextinction retrieval test without producing reductions in fear during extinction training, it is most likely that fluoxetine selectively promoted the consolidation of the extinction memory and not its acquisition [62]. Taken together, there is consensus that chronic fluoxetine treatment increases fear extinction or even erases the fear memory, but the behavioural as well as the neurobiological mechanisms appear to differ across studies. 

## 6. 5-HT Receptor Ligands

SSRIs as well as anxiolytic and antipsychotic agents exert their effects through one or more of the 17 5-HT receptor subtypes, particularly of the 5-HT_1_, 5-HT_2_, and 5-HT_3_ classes. These receptors are abundantly expressed in, amongst others, the amygdala and hippocampus [21, 63], where 5-HT_2_ and 5-HT_3_ agonists significantly increase the neuronal discharge rate and 5-HT_1A_ receptor agonists inhibit the firing rate (Stein et al. [64]). These functional effects are reflected at the behavioural level, as described in the following.

### 6.1. Effects of 5-HT_1A_Receptor Ligands on Fear Conditioning and Conditioned Fear Expression

The 5-HT_1A_ receptor is the key target of serotonergic anxiolytic agents, which function as partial or full agonists at this receptor [65]. In this context, Stiedl and coworkers [66] showed that postsynaptic 5-HT_1A_ receptor mediate, contextual fear, at the level of conditioning. Thus, pretraining intrahippocampal injections of 8-OH-DPAT caused a severe deficit in contextual fear when tested 24 hr after training. Intrahippocampal WAY 100635 (5-HT_1A_ antagonist) blocked the impairment caused by intrahippocampal but not subcutaneous 8-OH-DPAT, indicating the involvement of extrahippocampal 5-HT_1A_ receptors in fear conditioning. These data suggest that the deficits in fear conditioning induced by 8-OH-DPAT are a result of postsynaptic 5-HT_1A_ receptor activation that interferes with learning processes operating at acquisition but not consolidation. Furthermore, the dorsohippocampal 5-HT_1A_ receptors play an important but not exclusive role in the limbic circuitry subserving contextual fear conditioning. Koseki and colleagues [67] showed that adult rats that previously were exposed to five footshocks at postnatal day 21 to 25 exhibited impaired contextual fear extinction and that this effect was prevented by pretreatment with the 5-HT_1A_ receptor agonist tandospirone. Possibly, this effect may be attributed to an inhibitory influence on fear conditioning, leading to a stronger impact of the competing extinction memory.

5-HT_1A_ receptor agonists not only activate postsynaptic 5-HT_1A_ receptors but also presynaptic 5-HT_1A_ receptors. This distinction is relevant, as they have different functions in regulation of the serotonergic system. Presynaptic 5-HT_1A_ receptors serve as inhibitory autoreceptors and are found in the raphe nuclei, where they exert an inhibitory influence of the firing rate of serotonergic neurons [68, 69]. Overall, studies have shown that 5-HT_1A_ receptor agonists decrease contextual fear [70–72]. To elucidate whether this effect is mediated by pre- and/or postsynaptic 5-HT_1A_ receptors, Youn et al. [73] tested the effect of the 5-HT_1A_ full agonist and the selective presynaptic 5-HT_1A_ receptor agonist S15535 that has only weak effects at postsynaptic 5-HT_1A_ receptors on the expression on contextual fear. It was found that 8-OH-DPAT dose-dependently impaired the recall of contextual fear memory, which was mimicked to a lower extent by S15535. The weaker effect of S15535 compared to 8-OHDPAT suggests that particularly postsynaptic 5-HT_1A_ receptors are involved in the recall of fear memories. Yet, Borelli KG [74] showed that stimulation of 5-HT_1A_ autoreceptors in the median raphe nucleus with local injections of 8-OH-DPAT either before training or testing sessions conducted 2 or 24 h afterconditioning reduced contextual freezing, implying that a role for presynaptic 5-HT_1A_ receptors cannot be ignored. In further support of a contribution of postsynaptic 5-HT_1A_ receptors to the expression of contextual fear, it was reported by Li et al. [75] that bilateral microinjections of flesinoxan, another selective 5-HT_1A_ receptor agonist, into the hippocampus and amygdala decreased the expression of contextual fear, while injections into the medial prefrontal cortex did not. Moreover, flesinoxan infused either into the dorsal raphe nucleus or the median raphe nucleus did not affect startle potentiation, whereas bilateral infusion of flesinoxan into the CeA dose-dependently blocked fear-potentiated startle responses [76]. Further evidence for a greater contribution of post- compared to presynaptic 5-HT_1A_ receptors in the expression of contextual fear is derived from a study showing that infusion of 8-OHDPAT into the median raphe nucleus decreased contextual fear without an effect on fear-potentiated startle. When 8-OHDPAT was injected into the dorsal hippocampus both contextual conditioned freezing and fear-potentiated startle were reduced [77]. In the hippocampus, 5-HT_1A_ receptor agonism decreases the firing of hippocampal CA1 pyramidal neurons [78], which may explain the inhibitory effect of 5-HT_1A_ receptor agonism on contextual conditioned freezing. In sum, these rather consistent findings suggest that postsynaptic, and to a lesser extent presynaptic, 5-HT_1A_ receptors exert an inhibitory effect on both fear conditioning and the expression of contextual fear and fear-potentiated startle. 

5-HT_1A_ (ant)agonists not only modulate conditioned freezing on their own, but also modulate the effects of SSRIs. Thus, coadministration of a (sub)effective 5-HT_1A_ receptor agonist augmented SSRI-induced inhibition of the expression of contextual fear [52, 72]. Yet, Muraki and colleagues ([48] reported that citalopram (applied 24 hours after conditioning and 4 hrs before the test) significantly reduced conditioned freezing, an effect that was potentiated by coadministration of the 5-HT_1A_ receptor antagonist WAY 100,635, but not the 5-HT_1B/1D_ antagonist GR 127,935. These data suggest that 5-HT_1A_ receptor blockade facilitates the anxiolytic effect of citalopram, in line with the hypothesis that 5-HT_1A_ autoreceptor activation antagonizes the SSRI-mediated increases in 5-HT levels in the brain. Vice versa, chronic SSRI pretreatment increased the potency of a 5-HT_1A_ receptor agonist to inhibit contextual conditioned freezing [73]. Given that chronic SSRI treatment leads to desensitization of pre- and postsynaptic 5-HT_1A_ receptors [79] and that postsynaptic 5-HT_1A_ receptors and to a lesser extend presynaptic 5-HT_1A_ receptors mediate the inhibition of contextual fear, this cross-sensitization suggests that SSRIs exert their effects on contextual fear mainly through both pre- and postsynaptic 5-HT_1A_ receptors. 

### 6.2. Effects of 5-HT_2_ Receptor Ligands on Conditioned Fear Expression and Extinction

In the cortex, the 5-HT_2A_ receptor plays a central role in various learning and memory processes, including emotional memory. Functionally, activation of 5-HT_2A_ receptors enhances cortical presynaptic glutamate release [80], and humans carrying a polymorphism in the 5-HT_2A_ receptor gene show impaired consolidation of explicit memory [81]. The 5-HT_2A_ receptor also implicated fear conditioning and extinction, as was shown by Zhang and coworkers [82]. Specifically, they showed that the 5-HT_2A_ receptor agonist TCB-2 (1.0 mg/kg, i.p.) injected before the extinction facilitated the acquisition of extinction of cued fear memory, an effect that was delayed by the administration of the 5-HT_2A_ receptor antagonist MDL 11,939 (0.5 mg/kg, i.p.). Furthermore, the postconditioning administration of TCB-2 enhanced contextual and cued fear memory, whereas stimulation or blockade of 5-HT_2A_ receptors did not affect the encoding or retrieval of conditioned fear memory. These data showed that the 5-HT_2A_ receptor facilitates both the consolidation of cued fear memory and the consolidation of extinction memory. However, 5-HT_2A_ receptor knockout mice did not show changes in fear conditioning [83]. 5-HT_2C_ receptors, which are the targets of antidepressant (mirtazapine, agomelatine) and antipsychotic drugs, are also widely expressed in the cortex. Unlike the 5-HT_2A_ receptors, they are located on GABAergic interneurons in the PFC [84]. 5-HT_2C_ receptors may function in a negative feedback loop involving reciprocal interactions between GABAergic and serotonergic neurons in these regions. Furthermore, they are likely to reduce excitatory glutamate transmission, which thus opposes the function of 5-HT_2A_ receptors. The involvement of 5-HT_2C_ receptors in the expression of conditioned fear was shown by Burghardt et al. [49]. They showed that, after drug-free fear conditioning, a single pretesting injection of the SSRIs citalopram or fluoxetine significantly increased cued fear expression. This SSRI-induced enhancement in fear expression was not blocked by tropisetron, a 5-HT_3_ receptor antagonist, but was blocked by SB 242084, a specific 5-HT_2C_ receptor antagonist. This suggests that whereas activation of 5-HT_2A_ receptors facilitates the consolidation of cued fear and extinction memories, inactivation of 5-HT_2C_ receptors facilitate the retrieval of the cued fear memory. However, in the fear-potentiated startle test SB 24204 had no effect [85]. Possibly the effects of 5-HT_2C_ ligands are task dependent. 

### 6.3. Effects of 5-HT_3_ ****and 5-HT_7_ Receptor Ligands on Fear Conditioning and Expression

Furthermore, it has been reported that 5-HT_3_ receptor overexpression mice displayed increased contextual fear conditioning [86], but not cued fear conditioning and contextual and cued fear extinction [87]. The increased contextual fear learning was also seen as a tendency in 5-HT_3_ knockout mice, which displayed increased fear conditioning as well [88]. In line, tropisetron and ondansetron, selective 5-HT_3_ receptor antagonists, decreased the expression of contextual fear and contextual potentiated startle [89, 90]. These data suggest that the 5-HT_3_ receptor is specifically involved in contextual fear. Finally, 5-HT_7_ knockout mice were found to show impaired contextual fear conditioning along with decreased long-term synaptic plasticity within the CA1 region of the hippocampus [91].

## 7. Serotonin Transporter and Monoamine Oxidase A Gene Variation 

The serotonergic system can also be influenced by variations in serotonergic genes. The most widely studied serotonergic gene variation is the 5-HTTLPR, a 43 bp insertion/deletion polymorphism in the promoter region of the 5-HTT gene. The low activity s-allelic variant is associated with decreased transcription of the gene compared to the long (l) allele as well as reduced 5-HTT function. Presumably, this leads to increased extracellular 5-HT levels. It is well established that the s-allele is associated with trait anxiety [31, 92], due to amygdala hyperactivity in response to pictures of fearful faces [36] and a functional uncoupling between the PFC and amygdala [38]. S-allele carriers also demonstrate a smaller volume of the hippocampus [93, 94]. Recently, another 5-HTT polymorphism has been detected, the STPP/rs3813034 serotonin transporter polyadenylation polymorphism. Polyadenylation is a posttranscriptional modification of the 3′ end of the transcript that occurs in the majority of protein-coding mRNAs and differs between individuals. The STPP/rs3813034 serotonin transporter polyadenylation polymorphism influences the balance of two polyadenylation forms in the brain [95], of which G-allele carriers have a reduced fraction of 5-HTT mRNA containing the distal polyadenylation sequence and probably reduced 5-HTT mRNA levels [96].

MAOA (monoamine oxidase A) is responsible for the breakdown of serotonin, norepinephrine, and to a lesser extent dopamine. The variable number of tandem repeats polymorphism (MAOA-uVNTR) of the MAOA gene consists of genotypes with low (MAOA-L) and high (MAOA-H) activity [97]. Hence, like the 5-HTTPR s-allele, the MAOA-L variant is expected to be associated with increased extracellular 5-HT levels. Furthermore, MAOA-L carriers show amygdala hyperreactivity in response to pictures of fearful faces [98], with concomitant prefrontal hyporeactivity [98]. Yet, unlike the 5-HTTLPR s-allele, MAO-L has been particularly associated with higher trait aggression [99]. 

### 7.1. Effects of Serotonin Transporter Gene Variation on Fear Conditioning, Expression, Extinction, and Extinction Recall

In line with the above-described changes in the fear circuit of 5-HTTLPR s-allele carriers, it has been found that s-allele carriers displayed increased fear conditioning [100]. Furthermore, 5-HTTLPR s-allele carriers showed stronger fear-potentiated startle compared to l-allele homozygotes [101–103]. However, s-allele carriers showed no deficit in the extinction of fear after the offset of the fear-predicting CS [101]. Furthermore, s-allele subjects showed increased observational fear learning. Observational fear conditioning is a model of emotional learning by social means. Humans and other primates can vicariously learn to display fear to initially neutral stimuli after observing the emotional expression of a conspecific in which those neutral stimuli were paired with shocks. This process involves the amygdala, like normal fear conditioning [104]. Crisan and coworkers [105] reported that s-carriers tended to display enhanced autonomic responses when they observed a model being submitted to an aversive event, knowing that the same treatment awaits themselves, and showed increased autonomic responses to CS+ when they were subsequently placed in an analogous situation. A recent study demonstrated that the common STPP/rs3813034 polymorphism is associated impaired retention of fear extinction memory and heightened anxiety and depressive symptoms [106]. Thus, effects of the 5-HTTLPR and STPP polymorphisms seem comparable, but they affect distinctive parts of the fear conditioning/extinction process. At the neural level it was found that subjects carrying the short allelic variant of the 5-HTTLPR polymorphism showed elevated fear-CS elicited activity within the fear network (amygdala, insula, thalamus, occipital cortex) [107]. Furthermore, a recent neuroimaging study reported that trait anxiety was positively correlated with the magnitude of amygdala reactivity to a fear CS [108]. In contrast, Lonsdorf et al. [102] reported increased startle responses in s-allele compared to homozygous l-allele carriers, whereas no differences in conditioned SCRs emerged. 

5-HTT knockout mice and rats are well accepted as rodent models of the 5-HTTLPR s-allele [109, 110] and the STPP/rs3813034 polymorphisms [106]. In 5-HTT knockout rats it has been found that cue-induced fear extinction is delayed [111, 112]. This was also found in 5-HTT knockout mice [113], although other 5-HTT knockout mouse studies revealed that the mice do no differ in cue-induced fear extinction, but rather show impaired recall of the extinction memory [114, 115]. In contrast to the increased cued conditioned freezing, no changes have been observed in contextual conditioned freezing in 5-HTT knockout mice [116]. Although all studies so far indicate that cued conditioned fear is increased in 5-HTT knockout mice and rats, it is up to date unclear why either fear extinction or the extinction memory recall is affected by 5-HTT knockout in rodents. Differences are also noted between 5-HTTLPR and STPP/ rs3813034 effects. Possible explanations are species or strain-specific effects, or experimental procedures. 

### 7.2. Effects of Monoamine Oxidase A Gene Variation on Fear Conditioning, Expression, Extinction, and Extinction Recall

Whereas the 5-HTTLPR s-allele was found to enhance fear conditioning and tended to delay fear extinction, no significant effects were found for the low activity variant of the MAOA polymorphism. Yet, there was a trend for increased fear conditioning, as was found for the 5-HTTLPR s-allele [100], which is in line with the idea that both are associated with increased extracellular 5-HT levels. Possibly, a power or gender effect played is in the discrepancy between the 5-HTTLPR and MAOA effects, or the effect of MAOA on norepinephrine levels interfered with the enhanced fear conditioning.

The MAOA low activity variant can be mimicked by MAOA knockout mice. Whereas the effects of the MAOA polymorphism in humans were marginal, MAOA-deficient mice exhibited significantly enhanced classical fear conditioning, contextual conditioned freezing, and cued conditioned freezing. Yet, extinction was not clearly visible in the animals, and extinction recall was not measured [117]. Nevertheless, the effects of MAO-A knockout, compared to 5-HTT knockout, seem to be more diffuse. Perhaps this can be explained by the fact that MAOA knockout mice show increased extracellular levels of both serotonin and norepinephrine in the frontal cortex [117].

## 8. Serotonin Depletion

A final approach to study the role of 5-HT in fear conditioning is 5-HT depletion. There are a couple of approaches to reduce central 5-HT levels. One approach is acute tryptophan depletion (ATD). Tryptophan is the precursor of 5-HT and an essential amino acid. Tryptophan is depleted by ingestion of an amino acid mixture that does not contain this essential amino acid but does include other large neutral amino acids [118]. The amino acid load increases protein synthesis in the liver and increases competition for transport across the blood brain barrier, with both factors decreasing tryptophan availability in the brain. The advantage of this approach, especially in humans, is that effects are reversible. Yet, the 5-HT depletion is not confined to a particular brain region. 5-HT can also be depleted by systemic or local injection of parachlorophenylalanine (pCPA), which inhibits Tph activity [119], or by local injection of the neurotoxin 5,7-hydroxytryptamine (5,7-DHT). This toxin is taken up into cells via the 5-HTT and noradrenaline transporter and at appropriate doses selectively destroys these neurons [120]. When 5,7-DHT is injected into the ventricles 5-HT depletion will be depleted globally. Finally, given that the development of serotonergic neurons in the raphe nuclei is triggered by a cascade of transcription factors, knockout of one of the most essential ones, like *Lmx1b *(LIM homeobox transcription factor 1 beta), leads to global 5-HT depletion as well [121, 122]. 

### 8.1. Effects of Tryptophan Depletion and 5-HT Synthesis Inhibition on Fear Expression

In healthy human subjects, ATD significantly increased long-duration context-potentiated startle but had no effect on short-duration fear-potentiated startle. When indexing context-potentiated started as anxiety, and fear-potentiated started as fear, these results suggest that 5-HT depletion in humans selectively increases anxiety but not fear [30]. In mice, it was found that ATD impaired the formation of contextual fear memory, whereas the expression of cued fear memory was unaffected [123]. Yet, Hughes and Keele [124] showed that pCPA treatment selectively enhanced fear-potentiated startle in individually housed rats. An early study reported that acute fenfluramine and p-chloroamphetamine (PCA)—agents that induce 5-HT release through reverse 5-HTT function—treatment impaired the expression of conditioned fear, and pCPA treatment was unable to reverse this effect [125]. This implies that different 5-HT stores are involved in the action of PCA and pCPA and thus that the mechanisms underlying 5-HT depleting agents and 5-HTT inhibitors may be dependent on intracellular 5-HT dynamics, which remains to be resolved. 

### 8.2. Effects of Neurotoxin-Mediated Serotonin Depletion on Fear Expression

Using the neurotoxin approach, one study reported that intraventricular injections with 5,7-DHT in three-day-old rats did not alter contextual fear during adulthood [126]. Because there is high consensus that the prefrontal cortex controls emotion and that particularly the serotonergic modulation of prefrontal function is involved, Lehner and colleagues (2008) used 5,7-DHT to selectively deplete 5-HT in the PFC. Using the contextual fear conditioning paradigm, it was found that 5,7-DHT injected rats altered contextual fear, which was dependent on the initial footshock sensitivity of the animals: in high sensitive rats the serotonergic lesion significantly decreased contextual fear (along with enhanced c-Fos expression in the dorsomedial prefrontal area, and increased the concentration of GABA in the BLA), whereas in low sensitive animals the serotonergic lesion increased contextual freezing (along with increased c-fos expression in the BLA and CeA and a decreased GABAergic tone in the BLA). Possibly, the high sensitive rats were prepared for high stress levels, which allowed them recruit an inhibitory system during the contextual fear test upon 5-HT depletion. This illustrates the importance of individual differences in sensitivity to stress in fear conditioning processes.

### 8.3. Effects of Genetically Mediated Serotonin Depletion on Fear Conditioning and Expression

Genetic deletion of 5-HT in the mouse brain, which was achieved by inactivating the serotonergic transcription factor Lmx1b selectively in the raphe nuclei of the brainstem, enhanced contextual fear conditioning and expression. Particularly the retrieval of contextual fear memory was greatly enhanced in these mice. Furthermore, bath application of 5-HT in the 5-HT-deficient mice restored footshock-induced alterations of hippocampal synaptic plasticity. Since this enhancement could be prevented by intracerebroventricular administration of 5-HT, it seems that 5-HT levels per se, rather than neurodevelopmental, or compensatory mechanisms, are involved in this effect of 5-HT depletion [127, 128]. 

## 9. Discussion and Conclusions

Clearly, 5-HT plays an essential role in conditioned fear, but do the studies reviewed here provide a general view on the precise nature of 5-HT's role? The insights that the studies provide are as follows.

- Acute SSRI treatment facilitates fear conditioning.
- Acute SSRI treatment reduces the expression of contextual fear and increases the expression of cued fear.
- Chronic SSRI treatment reduces both contextual and cued fear.
- Activation of postsynaptic and to a lesser extent presynaptic 5-HT_1A_ receptors leads to inhibition of the acquisition and expression of contextual fear.
- Activation of 5-HT_2A_ receptors facilitates the consolidation of cued and contextual fear and extinction memories.
- Inactivation of 5-HT_2C_ receptors facilitates the retrieval of the cued fear memory.
- The 5-HT_3_ receptor mediates contextual fear.
- Genetic 5-HTT downregulation is associated with increased fear conditioning, impaired cued fear extinction, or impaired extinction recall.
- Genetic MAOA downregulation is associated with increased fear conditioning and increased expression of cued and contextual fear.
- ATD affects contextual (sustained) anxiety, whereas pCPA treatment affects cued fear.
- 5,7 DHT affects contextual fear, as function of preexisting sensitivity to footshock.
- Genetically induced 5-HT depletion increased fear condition and the expression of contextual fear.

**Figure 3:**
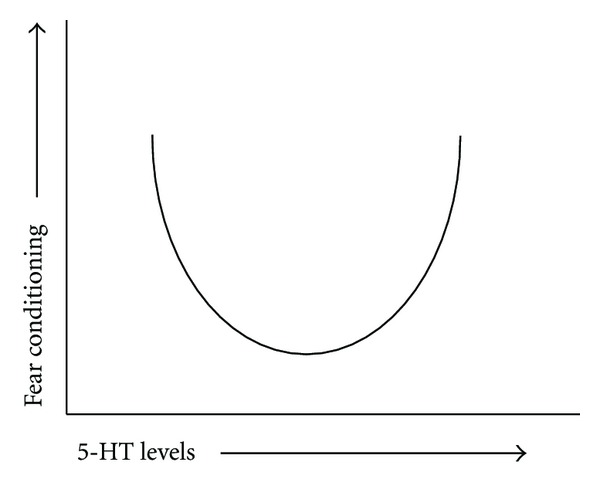
Suggested U-shaped relationship between 5-HT levels and the extent of fear conditioning. Available studies suggest that both too high (e.g., 5-HTTLPR s-allele, 5-HTT knockout, MAO-L, acute SSRI) and too low (e.g., Lmx1b knockout, ATD, pCPA) 5-HT levels increase associative learning and strengthen the association between the CS and the shock.

In terms of the relationship between 5-HT levels and fear conditioning, the data show that both increases in 5-HT levels (acute SSRI (a); 5-HTT (h); and MAOA (i) downregulation) genetically induced 5-HT depletion (l)) are associated with increased fear conditioning, the process during which the subjects acquire an association between the cue (and context) and the footshock. Possibly, this indicates that there is an U-shaped relationship between 5-HT levels and (fear) conditioning, such that both too low and too high 5-HT levels facilitate this process, whereas normal range of 5-HT levels is less effective (Figure 3). Given that fear conditioning is an adaptive response when there are threats (which is the case when subjects are exposed to shocks), it may be that extremes have a benefit in threatful situations. Interestingly, and inverted U-shaped relationship has been reported for the role of dopamine in working memory, such that both too low and too high dopamine levels impair this cognitive function [29]. This appears functionally opposite to that of 5-HT.

The idea that 5-HT facilitates associative learning processes like fear conditioning, and thereby impairs fear extinction or the recall of the extinction memory, fits well with the finding that behavioural control (active avoidance of shocks) goes along with inhibition of stressor-induced activation of 5-HT neurons in the dorsal raphe nucleus (for review see [129]). This inhibition is provided by the vmPFC efferents that terminate on GABA interneurons in the dorsal raphe nucleus [129]. Inactivation of the vmPFC takes away this inhibitory influence, which leads to increased fear learning [130].

The mechanism through which 5-HT mediates the expression of fear memory may involve the corticotropin-releasing factor (CRF) system in the raphe nuclei projecting to fear-related brain regions. Ohmura and coworkers ([57]) found that injection of the CRF_2_ receptor antagonist astressin 2B but not the CRF_1_ antagonist antalarmin into the dorsal raphe nucleus significantly suppressed the expression of conditioned freezing in the contextual fear-conditioning test. Furthermore, the CRF_2_ antagonist suppressed 5-HT release in the ventral hippocampus during fear memory retrieval. These findings show that 5-HT facilitates fear memory recall and that this effect is mediated by the CRF system. 

Another insight that emerges from this summary is that 5-HT acutely exerts opposite effects on contextual and cued fear (b). Since contextual fear is considered as more sustained long-duration and less specific fear than cued fear, and thereby anxiety rather than fear, the data suggest that different mechanisms are involved in anxiety and fear. The finding that acute SSRI treatment increases cued fear may be consistent with the clinical observation that symptoms worsen initially upon the start of the SSRI treatment, and has been attributed to the activation of 5-HT_1A_ autoreceptors in the raphe nuclei. The data that have been reviewed here reveal that pre- and postsynaptic 5-HT_1A_ receptors inhibit the expression of contextual fear [4], which may fit the findings that 5-HT reduces the expression of contextual fear. However, although data concerning the effects of 5-HT_1A_ receptor manipulations on cued fear are limited, it has been shown that activation of the 5-HT_1A_ receptor increases fear-potentiated startle [76]. This appears somewhat puzzling with the classical view that acute SSRI treatment stimulates the 5-HT_1A_ autoreceptor and thereby decreases raphe firing [42]. Perhaps, local increases in terminal regions due to 5-HTT inhibition compensate this effect. Yet, when SSRIs are administered chronically, they reduce both contextual and cued fear (c). Given that SSRIs are typically taken chronically, rather than acutely, in the treatment of depression and anxiety disorders (as well as other disorders like autism spectrum disorders and obsessive compulsive disorder), treatment outcome is particularly beneficial for patients.

This is also what can be concluded for anxiolytic agents, like buspirone, flesinoxan, and tandospirone, which function as partial or full 5-HT_1A_ agonists. 5-HT_2A_ receptors are the target of antipsychotic drugs like risperidone, olanzapine, clozapine, and aripiprazole [131], and 5-HT_2A_ receptors also mediate the hallucinogenic effects of LSD [132]. Because antipsychotics inhibit the 5-HT_2A_ receptor, the findings that 5-HT_2A_ receptor activation enhances contextual and cued fear (e) fit with the fact that psychoses can be associated with excessive fear sensations [133] and support the validity of the contextual/cued fear paradigm. Also the fact that chronic SSRI exposure downregulates 5-HT_1A_ and 5-HT_2A_ receptors [128, 134] fits with the findings that 5-HT_1A_ receptor activation (d) and 5-HT_2A_ inactivation decrease conditioned fear (e).

5-HT_2A_ and 5-HT_2C_ receptors are known to exert opposite effects on dopamine (DA) release. For example, the 5-HT_2C_ receptor antagonist M100907 was found to decrease DA efflux in the NAc, and the 5-HT_2C_ antagonist SB242084 increased DA efflux [135–138]. This is reflected by opposite effects on impulsivity and compulsivity [6], and cocaine self-administration and extinction of cocaine seeking behaviour [139]. The few studies reviewed here suggest that 5-HT_2A_ and 5-HT_2C_ receptors also have differential, and perhaps opposite, effects on fear consolidation, and retrieval of fear memory (e, f). The underlying mechanism is unclear as suggested (Section 6.3), 5-HT_1C_ receptors are likely to reduce excitatory glutamate transmission trough activation of GABAergic neurons in the prefrontal cortex and raphe nuclei, whereas activation of 5-HT_2A_ receptors has opposite effects. Inactivation of 5-HT_2A_ receptors and activation of 5-HT_2C_ receptors may increase or disinhibit glutamatergic neurotransmission and thereby facilitate the consolidation or retrieval of the fear memory. Yet, more research is needed to understand whether indeed and why different processes (consolidation versus retrieval) are affected by 5-HT_2A_ and 5-HT_2C_ receptors. 

**Figure 4:**
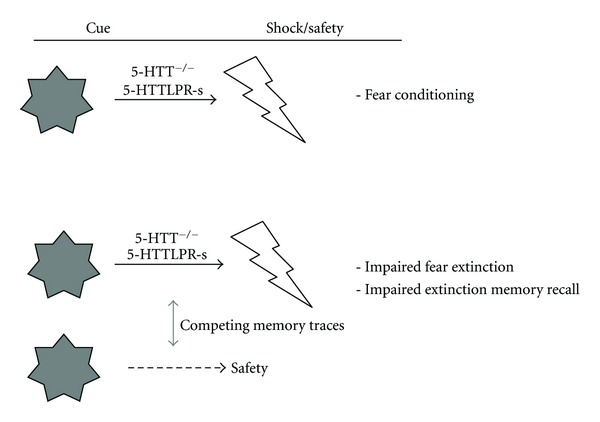
Potential explanation for the differential effects of genetic 5-HTT downregulation in humans and rodents on fear conditioning, expression, extinction, and extinction recall. 5-HT may strengthen associative learning processes, and thereby the association between a CS and shock (CS-shock memory trace). During extinction subjects acquire a second memory, namely, between the CS and safety (CS-safety memory trace). It depends on the competition between the two, what the behavioural expression of subjects will be. Because 5-HTT^−/−^ knockout rodents and 5-HTTLPR s-allele carriers are sensitive to motivationally relevant stimuli, the CS-shock memory trace may be “stronger” than the CS-safety memory trace, and thus prevail. The specific environmental conditions (e.g., housing, novelty) may determine at which phase (conditioning, expression, extinction, extinction recall) this CS-shock memory is expressed.

Lastly, whereas 5-HTT downregulation in both humans and rodents is consistently associated with increased anxiety, it is remarkable that different processes are affected across studies (h). Thus the 5-HTTLPR s-allele has been associated with increased fear conditioning, and a tendency towards impaired extinction. Whether 5-HTT knockout rats show increased fear conditioning has not been assessed thus far, but they do show a clear impairment in fear extinction. This seems to correspond to the 5-HTTLPR s-allele finding. Yet, 5-HTT knockout mice as well as carriers of the STPP/rs3813034 polymorphism show mostly an impairment in the recall of the extinction memory (see Section 7.1). Given that 5-HTT knockout rats quickly acquire conditioned responses [111, 140–142], that different types of memories are acquired in the fear conditioning/extinction, and that there may be a competition between these memories, I postulate that the most prevalent memory may determine the behavioural response of the animals [111]. Since 5-HTTLPR s-allele carriers and 5-HTT knockout rodents are highly sensitive to environmental influences [35], including those in the fear conditioning/extinction test, it is possible that specific test conditions determine which memory is most prevalent or that environmental conditions (e.g., type of rearing/housing) determine which memory is most relevant under the given circumstances and thereby is given priority (Figure 4). Systemic changes in experimental setup may be helpful to clarify this issue. 

In sum, the studies I reviewed here indicate that 5-HT is a strong modulator of conditioned fear as well as that the relationship between 5-HT levels and various fear conditioning processes is not straightforward. On top of this, the complexity of the serotonergic system and the 17 distinctive 5-HT receptor subtypes a tight control over conditioned fear through pharmacological agents or genetic manipulations is still difficult. Nonetheless, available studies provide a rather consistent view on the role of, for instance, 5-HT_1A_ and 5-HT_2A_ receptors. Given that the expression of these receptors differs across individuals, for instance, in 5-HTTLPR s-allele carriers and 5-HTT^−/−^ rodents [110, 143], an outstanding question is the efficacy of for example, 5-HT_1A_ receptor ligands in individuals characterized by an inherent downregulation of these receptors. Addressing this question may reveal that alternative treatments may be needed for those individuals that are less responsive to classical anxiolytic agents. 

## Abbreviations/Glossary

5,7-DHT: 5,7-Hydroxytryptamine (serotonergic toxin)
5-HIAA: 5-Hydroxyindoleacetic acid (metabolite of serotonin)
5-HT: Serotonin
5-HTT: Serotonin transporter
5-HTTLPR: Serotonin transporter-linked polymorphic region (polymorphism in human serotonin transporter gene)
5-HTP: 5-Hydroxytryptophan (intermediate product in serotonin synthesis)
ATD: Acute tryptophan depletion (method to reduce serotonin levels in the brain)
BLA: Basolateral amygdala
BNST: Bed nucleus of the stria terminalis
CE: Central nucleus of the amygdala
CR: Conditioned response (response to CS predicting US)
CRF: Corticotrophin releasing factor (stress-related neuropeptide)
CS: Conditioned stimulus (e.g., tone, light predicting US)
DA: Dopamine
EMG: Electromyography
IL: Infralimbic cortex (ventral part of medial PFC in rodents)
LA: Lateral amygdala
MAO: Monoamine oxidase A (breaking down serotonin)
PAG: Periaqueductal grey (region in brainstem responsible for fight/flight responses to fear)
PET: Positron emission tomography
pCPA: Parachlorophenylalanine (inhibitor of serotonin synthesis)
PL: Prelimbic cortex (dorsal part of medial PFC in rodents)
PTSD: Posttraumatic stress disorder
SCR: Skin conductance response (CR in humans in response to shock)
SSRI: Selective serotonin reuptake inhibitor (antidepressants, antianxiety agent; citalopram, escitalopram, fluoxetine, fluvoxamine, paroxetine, sertraline
Tph: L-tryptophanhydroxylase (rate-limiting enzyme for serotonin synthesis)
UR: Unconditioned response (response to US)
US: Unconditioned stimulus (e.g., shock, loud noise)
Vmat: Vesicular monoamine transporter (for storage monoamines in intracellular vesicles)
vmPFC: Ventromedial prefrontal cortex
VNTR: Variable number tandem repeat (type of genetic polymorphism).
